# The Iodide of Ammonium and Sodium in Syphilis

**Published:** 1872-04

**Authors:** 


THE IODIDES OF AMMONIUM AND SODIUM IN SYPHILIS.—Dr. Berkely Hill, in the *British Medical Journal* of the 23rd of December, strongly recommends the use of the iodides of sodium and ammonium in syphilis after the iodide of potassium has failed to produce its good effects. They produce the effect of iodine in many persons who have become nauseated by the potassa salt. Moreover, they contain less alkali per weight of the salt, and their alkali is less deteriorating to the blood than potash. The Doctor reports several cases in support of these statements.—*Ibid; Nashville Medical Journal.*

